# Review of Delusional Jealousy and Its Association with Sexual Dysfunctions

**DOI:** 10.1192/j.eurpsy.2024.1582

**Published:** 2024-08-27

**Authors:** S. A. Pinho, F. Leitão, J. R. Freitas, F. Coutinho

**Affiliations:** Hospital de Magalhães Lemos, Centro Hospitalar Universitário de Santo António, Porto, Portugal

## Abstract

**Introduction:**

Mental state changes can affect one’s sexual life, while sexual dysfunction can lead to relationship challenges. Delusional jealousy, also called Othello syndrome, involves a paranoid belief in a partner’s infidelity, leading to controlling and violent behaviors. It can manifest as a paranoid disorder, as a delusional symptom of psychiatric, neurological or other medical conditions, or as side effect of dopaminergic medication. Although its exact prevalence remains uncertain, it has been identified in 0.5-1.4% of psychiatry inpatients.

**Objectives:**

To describe sexual dysfunctions associated with delusional jealousy and to explore strategies for addressing these dysfunction.

**Methods:**

A non-systematic review of the literature available at PubMed was conducted using the keywords “Sexual Dysfunction” AND “Delusional Jealousy OR Othello Syndrome”.

**Results:**

A number of factors, including sexual dysfunction, can trigger or exacerbate delusional jealousy. This is especially true for middle-aged men who have a history of alcohol consumption, neurological or personality disorders. Individuals with sexual dysfunction experience feelings of insecurity, projecting these concerns onto their partners and suspecting extramarital relationships. On the other hand, sexual dysfunctions such as Hypoactive Sexual Desire Disorder, Female Sexual Arousal and Orgasmic Disorders, Erectile Dysfunction and Ejaculation Disturbance may occur as consequence of Othello Syndrome. Multiple factors contribute to these dysfunctions, including increased testosterone and cortisol levels, chronic alcohol use, comorbid psychiatric conditions and antipsychotics. There are reports of increased sexual desire, especially in cases of dementia.

**Conclusions:**

Although the evidence is limited and dated, it points to a bidirectional association between delusional jealousy and sexual dysfunction. Further studies are essential to determine the prevalence and types of sexual dysfunctions in Othello syndrome, and the causal relationship between them. Additionally, investigating gender differences is crucial, given the male-centric focus of existing studies. This research can contribute to clinical care by promoting the screening for sexual issues and their integration into delusional jealousy management.

**Disclosure of Interest:**

None Declared

